# A *Bacillus sphaericus* Based Biosensor for Monitoring Nickel Ions in Industrial Effluents and Foods

**DOI:** 10.1155/JAMMC/2006/83427

**Published:** 2006-06-01

**Authors:** Neelam Verma, Minni Singh

**Affiliations:** 1 Biosensor Development Laboratory Department of Biotechnology Punjabi University Patiala 147 002 India

## Abstract

A microbial-based biosensor has been developed based on enzyme inhibition bioassay for monitoring the presence of Ni(II) in real-time samples. The sensing element is immobilized *Bacillus sphaericus* MTCC 5100 yielding urease enzyme. The transducer is an ${\text{NH}}_{4}^{+}$ ion selective electrode in conjunction with a potentiometer. Heavy metals are potentially toxic to human beings. Nickel is associated with causing adverse health effects such as dermatitis and vertigo, in humans. Toxicity is manifested by affecting T-cell system and suppressing the activity of natural killer cells. Nickel finds applications in electroplating, coinage, electrodes, jewellery, alloys. The foods rich in Ni(II) are nuts, beans, oats, and wheat. The range of Ni(II) detection by the developed biosensor is 0.03–0.68 nM (0.002–0.04 ppb) with a response time of 1.5 minutes. For application, the Ni(II) effluent was procured from an electroplating industrial unit and was found to have a concentration of 100.0 ppm Ni(II). In foods, wheat flour sample was acid digested and Ni(II) was specifically complexed in the presence of other cations, and had an Ni(II) concentration of 0.044 ppm. The developed system has a reliability of 91.5% and 90.6%, respectively, for the samples and could possibly replace the existing conventional techniques of analysis.


Hindawi Publishing Corporation
Journal of Automated Methods and Management in Chemistry
Volume 2006, Article ID 83427, Pages 1–4
DOI 10.1155/JAMMC/2006/83427

### A *Bacillus sphaericus* Based Biosensor for Monitoring Nickel Ions in Industrial Effluents and Foods

**Neelam Verma and Minni Singh**

*Biosensor Development Laboratory, Department of Biotechnology, Punjabi University, Patiala 147 002, India*

Received 13 December 2005; Accepted 4 January 2006

A microbial-based biosensor has been developed based on enzyme inhibition bioassay for monitoring the presence of Ni(II) in real-time samples. The sensing element is immobilized *Bacillus sphaericus* MTCC 5100 yielding urease enzyme. The transducer is an $NH_4^+$ ion selective electrode in conjunction with a potentiometer. Heavy metals are potentially toxic to human beings. Nickel is associated with causing adverse health effects such as dermatitis and vertigo, in humans. Toxicity is manifested by affecting T-cell system and suppressing the activity of natural killer cells. Nickel finds applications in electroplating, coinage, electrodes, jewellery, alloys. The foods rich in Ni(II) are nuts, beans, oats, and wheat. The range of Ni(II) detection by the developed biosensor is 0.03–0.68 nM (0.002–0.04 ppb) with a response time of 1.5 minutes. For application, the Ni(II) effluent was procured from an electroplating industrial unit and was found to have a concentration of 100.0 ppm Ni(II). In foods, wheat flour sample was acid digested and Ni(II) was specifically complexed in the presence of other cations, and had an Ni(II) concentration of 0.044 ppm. The developed system has a reliability of 91.5% and 90.6%, respectively, for the samples and could possibly replace the existing conventional techniques of analysis.

Copyright © 2006 N. Verma and M. Singh. This is an open access article distributed under the Creative Commons Attribution License, which permits unrestricted use, distribution, and reproduction in any medium, provided the original work is properly cited.

### 1. INTRODUCTION

Monitoring the environment for the presence of compounds which may adversely affect human health and local ecosystems is a fundamental part of the regulation, enforcement, and remediation processes which are required to maintain a habitable environment [1]. People have always been exposed to heavy metals. The burning of fossil fuels containing heavy metals, the addition of tetraethyl lead to gasoline, and the increase in industrial application of metals have made heavy metal poisoning the major source of environmental pollution [2]. Heavy metals are hazardous to the ecosystem and are a serious danger to human populations because of their ability to accumulate [3].

Most metal ions can be detected in environmental samples using analytical methods such as inductively coupled plasma atomic spectrometry or mass spectrometry, flow injection atomic absorption or electrochemical methods that include ion selective electrodes, polarography, and other voltammetric electrodes [4]. The biosensor, a recent product of biotechnology, has attracted considerable attention as the potential successor to a wide range of analytical techniques [5, 6]. A biosensor is an analytical tool or system consisting of an immobilized biological material in intimate contact with a suitable transducer device that converts the biochemical signal into a quantifiable electrical signal [7]. Biosensors have the advantages of specificity, fast response times, portability, ease of use, and a continuous real-time signal. Furthermore, their biological base makes them ideal for toxicological measurement of heavy metals, while conventional techniques can only measure concentration [3]. In many monitoring situations biosensors can be expected to be the most cost-effective technology [8, 9].

Nickel is an essential trace element, which is an activator of a number of enzymes such as alkaline phosphatase and oxaloacetate decarboxylase. It finds extensive application in electroplating besides being used in hydrogenation of oils, in corrosion resistant alloys and in jewellery. It is also known to enhance insulin activity [8]. However, excessive nickel can cause toxicity which is manifested by affecting the T-cell system and suppressing the activity of natural killer cells [9]. Higher concentrations of nickel can cause cancer [10]. Nickel is normally analysed by X-ray absorption spectroscopy [11]. The technique, although highly precise, suffers from the disadvantages of high cost and the need for trained personnel. Keeping this in view, this work aims at the development of a microbial system to form a second-generation biosensor to allow a reliable, accurate, and cost-effective technology based on inhibition phenomenon to monitor the presence of nickel (Ni(II)) ions in industrial effluents and food samples.

### 2. MATERIALS AND METHODS

All chemicals were of analytical grade. The source of urease was a microorganism isolated from soil. An ion selective electrode (ISE) coupled to a Cyberscan benchtop potentiometer was used to sense an ammonium such as $NH_4^+$.

### 2.1. Biocomponent

The biocomponent of the biosensor was microbial-based. A urease yielding microbe was isolated from soil using microbiological techniques [12] and was identified by Microbial Type of Culture Collection (MTCC) and Gene Bank, Institute of Microbial Technology (IMTECH), Chandigarh, as gram +ve *Bacillus sphaericus* MTCC 5100. The microbe was cultivated in a media containing urea (2.5%), beef extract (1.0%), peptone (1.0%), and sodium chloride (0.5%) with a pH 7.0 under aerobic conditions at 25°C. To use the microbe as a biocomponent, the culture was harvested after 24 hour incubation and the pellet was retained, followed by immobilization of the microbe by physical adsorption onto Whatman number 1 filter paper, which was then dried and coupled to the body of the ion selective electrode.

### 2.2. Transducer

The transducer was a benchtop potentiometer (Cyberscan 2500) in conjunction with an $NH_4^+$ ion selective electrode (ISE code number EC-NH4-03) that detects the electrode potential developed across the membrane of the electrode when it comes into contact with the $NH_4^+$ ions released as a result of urea hydrolysis, forming a second-generation biosensor. The electrode potential developed across the membrane of the electrode when it comes in contact with $NH_4^+$ ions is related to the concentration of free $NH_4^+$ ions in solution by a form of the Nernst equation $E = E^0 + \mathrm{RT/nF} \ln a$.

### 2.3. Calibration of $NH_4^+$ ISE

Ammonium standard stock solutions were prepared with concentrations in the range $55.5 \times 10^{-3} - 55.5 \times 10^{-6}$ M. The ionic strength of all standards and samples was adjusted with ISA (ionic strength adjustor −5 M NaCl). 2 mL of ISA was added to every 100 mL of sample and standard solutions to maintain a background ionic strength of 0.1 M. For preparing the calibration curve, the $NH_4^+$ ISE was subjected to the ammonium standards and the mV were recorded.

### 2.4. Hydrolysis

The microbial urease causes the hydrolysis of urea into $NH_4^+$ and $HCO_3^-$. The $NH_4^+$ ions released are sensed by the $NH_4^+$ ISE. For hydrolysis, 33.3 mM urea in PBS pH 7.5 was used at a temperature of 25°C [13]:

$$NH_2CONH_2 + 2H_2O + H^+ \xrightarrow{\text{Urease}} 2NH_4^+ + HCO_3^-. \quad (1)$$

The enzyme assay for free and immobilized system was performed to study the residual activity, that is, the activity of the immobilized biocomponent retained in comparison to the free cell system.

### 2.5. Optimization of response time and linear range of Ni(II) detection

The detection of Ni(II) was based on the inhibition of the enzyme activity. There is increased inhibition with increasing concentrations of Ni(II) ions having a linearity between the concentration of metal ion and the percentage (%) inhibition caused by it. The response time of detection was optimized by studying the reaction at 30 second intervals and their corresponding percentage (%) inhibitions.

The % inhibition is calculated from the relative activity (RA) as follows:

$$\mathrm{RA} = \frac{\Delta\,\mathrm{mV}_{(\mathrm{inhibited})}}{\Delta\,\mathrm{mV}_{(\mathrm{uninhibited})}}, \quad \%\ \text{inhibition} = (1 - \mathrm{RA}) \times 100. \quad (2)$$

### 2.6. Application and reliability of the developed biosensor

The developed microbial biosensor was applied to an electroplating industrial effluent; and a wheat flour sample that was pretreated by acid digestion and specific Ni(II) complexation with dimethylglyoxime in the presence of other cations, and the reliability was checked by adding known amounts of the heavy metal to the samples and determining the concentration of the heavy metal ion based on % inhibition.

### 3. RESULTS AND DISCUSSION

Figure 1 shows the rod shaped cells of the urease yielding gram +ve *B. sphaericus* isolated from soil.

A calibration curve for $NH_4^+$ as prepared using various concentrations of ammonium ions is shown in Figure 2.

The curve exhibits a Nernstian slope of 57.9 mV at 25°C. With the prolonged use of the $NH_4^+$ ISE and with minor changes in environmental conditions there is a small variation in the slope value:

(i) slope mean: 55.07,
(ii) standard deviation: ±3.80,
(iii) coefficient of variation: 6.9%.

The enzyme assay for free and immobilized system was compared to study the activity of the immobilized biocomponent retained in comparison to the free cell system. The results in Figure 3 indicate that as hydrolysis proceeds, the substrate availability towards the microbial enzyme increases, also indicating the outward cell membrane-based location of the urease from the isolated strain.

The detection of Ni(II) was based on enzyme inhibition. The inhibition principle is based on the effect of metal ion Ni(II) on the microbial urease. The activity of urease is inhibited in the presence of Ni(II). The postulated mechanism of enzyme inhibition is based on the interaction of the metal ion

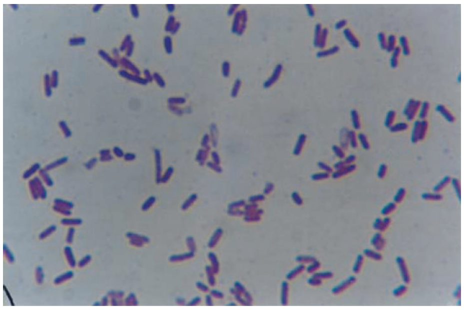

FIGURE 1: Urease yielding *B. sphaericus* MTCC 5100 isolated from soil.

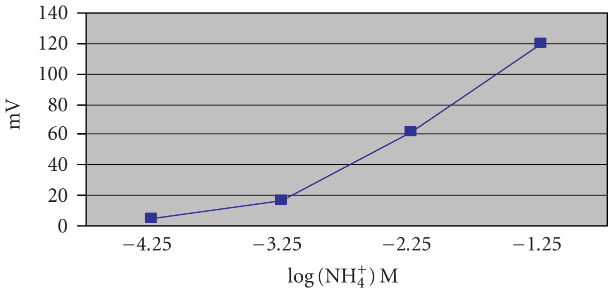

FIGURE 2: Calibration curve of NH$_4^+$ ISE.

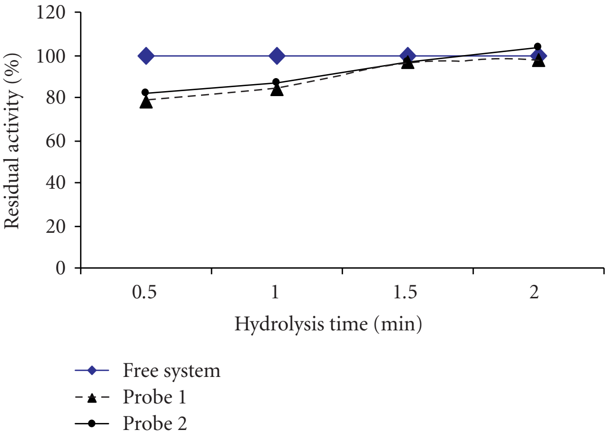

FIGURE 3: Residual activity of different biocomponents (probes) as a function of hydrolysis time.

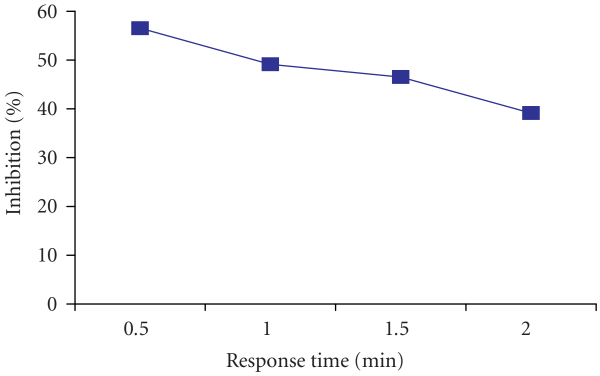

FIGURE 4: Optimization or response time for Ni(II) determination.

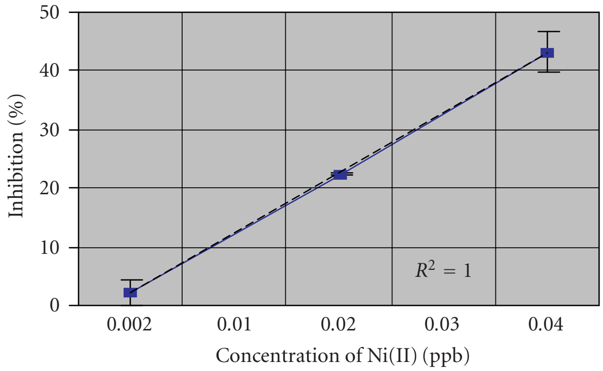

FIGURE 5: Linear range for Ni(II) determination (potentiometer).

TABLE 1: Reliability of the developed biosensor.

| | Effluent | Pretreated food sample |
|---|---|---|
| $\Delta$ mV$_{\text{(uninhibited)}}$ | 20.5 | 21.7 |
| $\Delta$ mV$_{\text{(inhibited)}}$ | 18.1 | 17.5 |
| % inhibition | 11.7% | 19.3% |
| Conc. (from graph) | 0.25 ng | 0.44 ng |
| $\Delta$ mV$_{\text{(uninhibited)}}$ | 20.6 | 22.0 |
| Conc. of sample (added) | 0.125 ng (0.25/2) | 0.22 ng (0.44/2) |
| Conc. of Ni(II) added | 0.125 ng (0.25/2) | 0.22 ng (0.44/2) |
| $\Delta$ mV$_{\text{(inhibited)}}$ | 18.4 | 17.3 |
| % inhibition | 10.7% | 21.3% |
| % reliability | 91.5% | 90.6% |

with exposed thiol or methylthiol groups of protein amino acids [14] and to the nonspecific binding of the heavy metal to the sulfhydryl groups [4] leading to a loss of its activity that is inhibition. The optimum response time was 1.5 minutes. It is the optimum time for all ranges of concentration. Figure 4 shows the optimization of response time for inhibition shown at 0.04 ppb (0.68 nM) Ni(II).

Figure 5 shows the inhibition caused by various concentrations of Ni(II) as recorded by the potentiometer on hydrolysis at pH 7.5 at a temperature of 25°C with 33.3 mM urea solution at a response time of 1.5 minutes. The linear range is 0.002–0.04 ppb (0.03–0.68 nM). For experimental purposes PVC ware was used as glass adsorbs metal ions.

The developed biosensor was applied to monitor the presence of Ni(II) in an electroplating industrial effluent and in pretreated food sample. The reliability was checked by adding known amount of the metal ion into the samples and then determining the % inhibition caused and hence the corresponding concentration from the linear scale developed. The concentration of Ni(II) in the effluent was 100 ppm and in the food sample was 0.044 ppm, with reliabilities of 91.5% and 90.6%, respectively, as shown in Table 1.

### REFERENCES

[1] K. R. Rogers and J. N. Lin, "Biosensors for environmental monitoring," *Biosensors and Bioelectronics*, vol. 7, no. 5, pp. 317–321, 1992.

[2] C. D. Klaassen, "Heavy metals and heavy metal antagonists," in *Goodman and Gilman's: The Pharmacological Basis of Therapeutics*, P. B. Molinkoff and R. W. Ruddon, Eds., pp. 1649–1650, McGraw-Hill, New York, NY, USA, 1996.

[3] M. J. Dennison and A. P. F. Turner, "Biosensors for environmental monitoring," *Biotechnology Advances*, vol. 13, no. 1, pp. 1–12, 1995.

[4] P. Corbisier, D. van der Lelie, B. Borremans, et al., "Whole cell- and protein-based biosensors for the detection of bioavailable heavy metals in environmental samples," *Analytica Chimica Acta*, vol. 387, no. 3, pp. 235–244, 1999.

[5] J. H. T. Luong, A. Mulchandani, and G. G. Guilbault, "Developments and applications of biosensors," *Trends in Biotechnology*, vol. 6, no. 12, pp. 310–316, 1988.

[6] N. Verma and M. Singh, "Biosensors for heavy metals," *BioMetals*, vol. 18, no. 2, pp. 121–129, 2005.

[7] M. Gronow, "Biosensors," *Trends in Biochemical Sciences*, vol. 9, no. 8, pp. 336–340, 1984.

[8] H. D. Belitz and W. Grosch, *Food Chemistry*, Springer, Berlin, Germany, 2nd edition, 1999.

[9] WHO-World Health Organization, in *A WHO Task Group on Environmental Health Criteria for Nickel*, E. Smith, Ed., p. 17, 1989.

[10] D. Parag, "Industrial effluents leak into Rajkot water table," *The Indian Express*, 14 July, 2000.

[11] K. J. Tiemann, J. L. Gardea-Torresdey, G. Gamez, et al., "Use of X-ray absorption spectroscopy and esterification to investigate Cr(III) and Ni(II) ligands in alfalfa biomass," *Environmental Science & Technology*, vol. 33, no. 1, pp. 150–154, 1998.

[12] R. E. Stanier, J. L. Ingraham, M. L. Wheelis, and P. R. Painter, "Gram positive Eubactria: unicellular endospore formers," in *General Microbiology*, pp. 475–494, Macmillan Press, London, UK, 5th edition, 1987.

[13] W. H. R. Shaw and D. N. Raval, "The inhibition of urease by metal ions at pH 8.9," *Journal of the American Chemical Society*, vol. 83, no. 15, pp. 3184–3187, 1961.

[14] T. K. Krawczyk, M. Moszczyn'ska, and M. Trojanowicz, "Inhibitive determination of mercury and other metal ions by potentiometric urea biosensor," *Biosensors and Bioelectronics*, vol. 15, no. 11-12, pp. 681–691, 2000.

